# Photochemical synthesis of silver nanoprisms via green LED irradiation and evaluation of SERS activity

**DOI:** 10.3762/bjnano.16.103

**Published:** 2025-08-26

**Authors:** Tuan Anh Mai-Ngoc, Nhi Kieu Vo, Cong Danh Nguyen, Thi Kim Xuan Nguyen, Thanh Sinh Do

**Affiliations:** 1 Nanotechnology Lab, Research Laboratories of Saigon Hi-Tech Park, Lot I3, N2 Street, Tang Nhon Phu Ward, Ho Chi Minh City 70000, Vietnam

**Keywords:** light-emitting diodes (LEDs), photochemical synthesis, silver nanoprisms, surface-enhanced Raman scattering (SERS), trisodium citrate

## Abstract

Silver nanoprisms (AgNPrs) are promising candidates for surface-enhanced Raman scattering (SERS) due to their strong localized surface plasmon resonance and sharp tip geometry. In this study, AgNPrs were synthesized through a photochemical method by irradiating spherical silver nanoparticle seeds with 10 W green light-emitting diodes (LEDs; 520 ± 20 nm) for various periods of time up to 72 h. The growth mechanism was investigated through ultraviolet–visible spectroscopy, field-emission scanning electron microscopy, X-ray diffraction, and transmission electron microscopy analyses, confirming the gradual transformation of spherical seeds into AgNPrs. Optimal conversion was observed after 72 h of irradiation, producing well-defined AgNPrs with an average size of 78 nm. The SERS activity of the AgNPrs was evaluated using 4-mercaptobenzoic acid as a probe molecule. Compared to spherical AgNPs, AgNPrs exhibited a significantly higher SERS enhancement factor of 1.15 × 10^6^, enabling detection limits down to 10^−9^ M. These findings demonstrate that green LED-mediated synthesis provides a simple, environmentally friendly route to fabricate high-yield AgNPrs with superior SERS capabilities, suitable for ultrasensitive chemical and biological sensing applications.

## Introduction

Anisotropic silver nanoparticles (ASNPs) have attracted increasing attention from research groups worldwide due to their potential applications in optical sensing, particularly in surface-enhanced Raman scattering (SERS) [1]. Among ASNPs, silver nanoprisms (AgNPrs) are of particular interest because of their broad absorption in the visible range (400–900 nm), enabling them to display a wide spectrum of colors such as yellow, red, orange, violet, green, and blue. This makes AgNPrs highly suitable for optical sensing applications [2].

The formation mechanism of AgNPrs in solution typically proceeds through three stages, namely, nucleation, seeding, and crystallization, with the crystallization phase being the slowest and rate-determining step of the entire process [3]. During this stage, small silver nanoparticles (seeds) can combine and evolve into anisotropic nanostructures through a chemical process in the presence of hydrogen peroxide (H_2_O_2_) and sodium borohydride, commonly referred to as the Mirkin method [4]. Recently, photochemical methods utilizing physical agents such as lasers [5], UV light [6], or LEDs [7–9] have gained attention due to their superior spatial and temporal control, high stability of the resulting AgNPrs and avoidance of environmentally unfriendly reducing agents [8,10].

The strong surface plasmon resonance (SPR) exhibited by AgNPrs significantly contributes to SERS enhancement by amplifying local electromagnetic fields. This makes AgNPrs ideal candidates for SERS-based sensing applications [1]. Numerous studies have focused on the fabrication of SERS-active substrates by depositing AgNPrs onto various solid supports such as glass [11], quartz, silicon, or aluminum foil [12]. A recent trend in the development of practical SERS substrates emphasizes flexibility and portability for field-deployable Raman detection. Accordingly, several groups have developed paper-based [13] or cotton-based [14] SERS substrates by impregnating them with silver nanostructures. While many efforts have been devoted to the synthesis of AgNPrs and evaluation of their SERS activity, limited studies have focused on the SERS performance of photochemically synthesized AgNPrs, particularly in 4-mercaptobenzoic acid (4-MBA) as a probe molecule.

In this study, AgNPrs were synthesized photochemically using green LED 10 W irradiation. Seeds were prepared using 1 mM AgNO_3_ and stabilized with a trisodium citrate (TSC)/AgNO_3_ molar ratio of 5. Physicochemical analyses confirmed the successful formation of AgNPrs with high yield. The SERS performance of the synthesized AgNPrs, evaluated using 4-MBA as a probe molecule, demonstrated significantly enhanced Raman signals compared to the initial AgNPs seeds, highlighting their potential applicability in SERS-based sensors.

## Results and Discussion

### Synthesis of seeds

**Figure 1:**
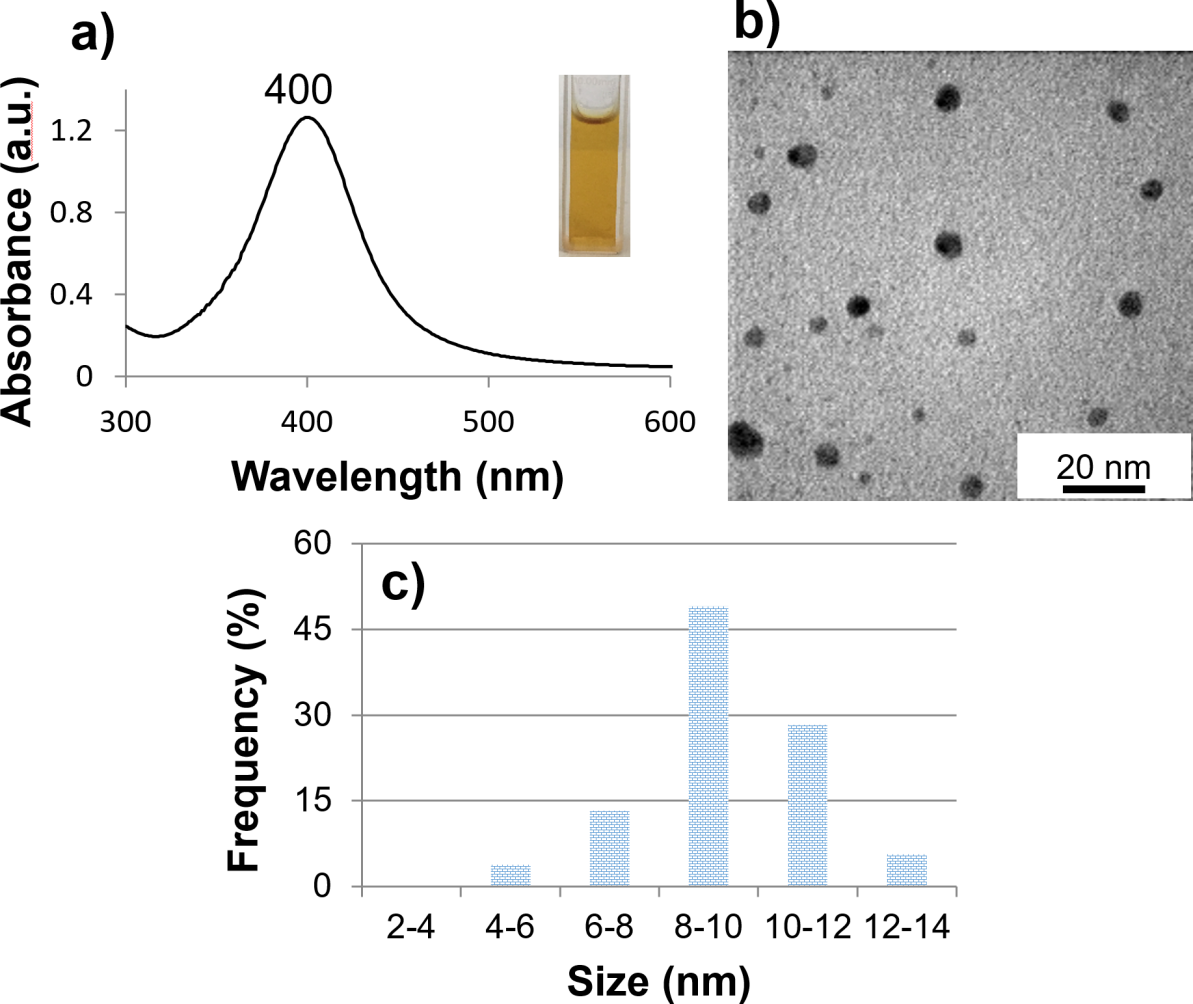
(a) UV–vis spectrum, (b) TEM image and (c) size distribution of seeds.

The UV–vis spectra of the AgNPs seed shown in the Figure 1a displayed a single peak at 400 nm, indicating the presence of spherical silver nanoparticles with small sizes, consistent with studies in the literature [4,6–7]. The sample exhibited the characteristic yellow color of AgNPs. The TEM image and size distribution in the Figure 1b,c showed that the seed predominantly contained spherical silver nanoparticles with a size of approximately 10 nm, in agreement with the UV–vis spectrum result.

### Manufacturing of AgNPrs

**Figure 2:**
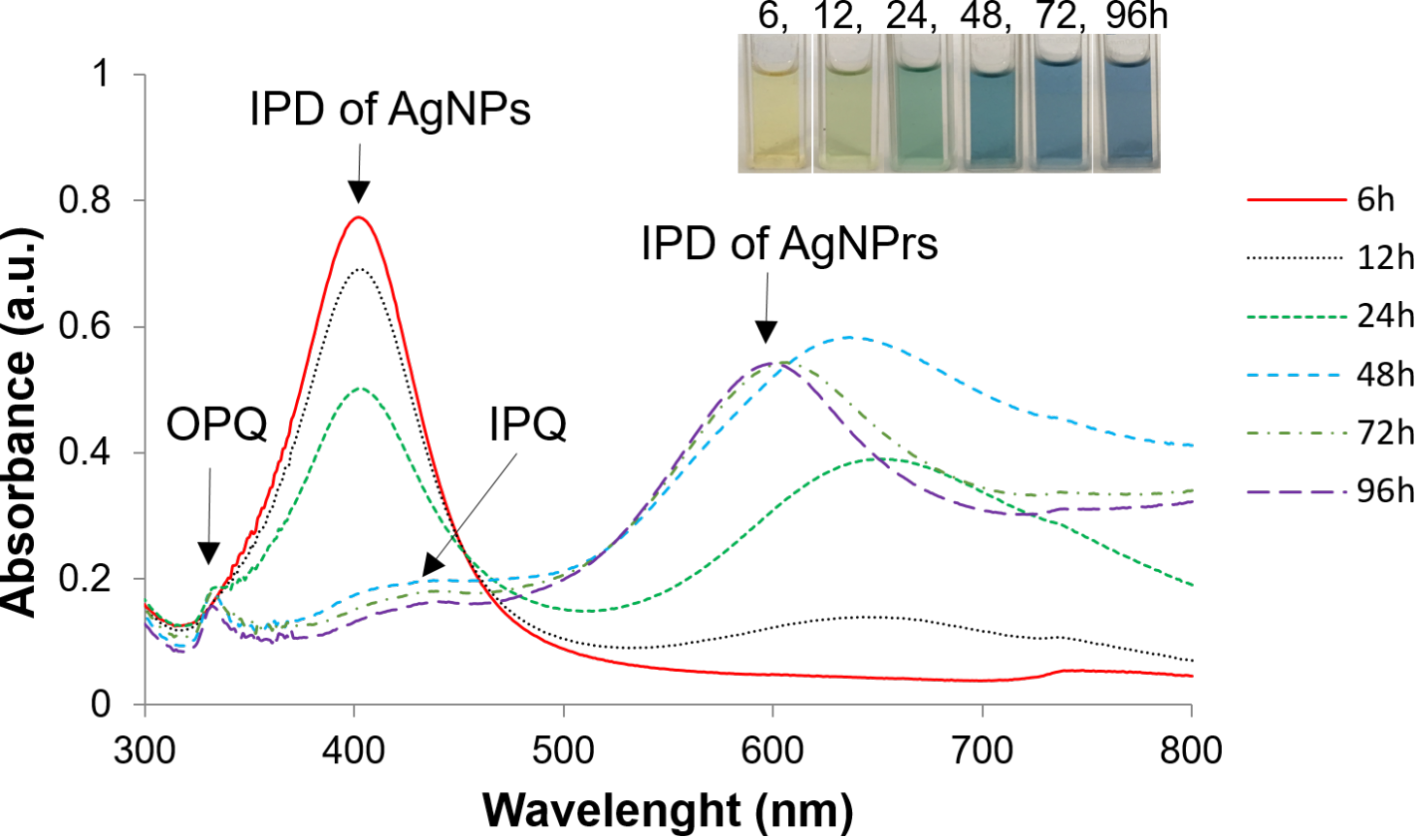
UV–vis spectra of silver nanoparticles at different LED irradiation times.

AgNPrs were synthesized in two steps, that is, (i) synthesis of AgNPs as seeds and (ii) irradiating the seeds with green LEDs to form AgNPrs with the aid of ʟ-arginine (L-A) [9]. Due to the different SPR properties between AgNPs and AgNPrs, the morphological transformation of silver nanostructures in sample can be observed through UV–vis spectra in Figure 2.

After 6 h of LED irradiation, the UV–vis spectrum exhibited a single absorption peak at 402 nm, characteristic of the in-plane dipole (IPD) resonance of spherical silver nanoparticles. The solution retained its yellow color, indicating that the seed morphology remained largely unchanged and that no formation of anisotropic silver nanostructures had occurred at this stage [4,7]. The IPD peak was still observed after 12 and 24 h but with progressively decreasing intensity, suggesting a reduction in the number of spherical silver seeds as the irradiation time increased.

After 12 h, in addition to the IPD peak at 405 nm, a broad shoulder began to emerge at approximately 650 nm. This shoulder became more prominent after 24 h, shifting slightly to 662 nm. The appearance of this absorption feature indicated the onset of anisotropic nanoparticle formation, such as silver nanoprisms [4–7].

After 48 h of LED irradiation, the IPD peak associated with spherical nanoparticles was nearly undetectable. Instead, the UV–vis spectrum displayed three distinct absorption peaks, that is, (i) an out-of-plane quadrupole (OPQ) peak at 333 nm, indicative of the nanoplate thickness; (ii) an in-plane quadrupole (IPQ) peak at 440 nm with weak intensity; and (iii) a strong in-plane dipole (IPD) peak at 654 nm, characteristic of silver nanoprisms. This observation is consistent with the characteristic UV–vis absorption spectra of AgNPrs reported in previous studies [2,4,7]. The simultaneous presence of these three plasmonic modes, along with the disappearance of the spherical IPD peak, confirmed that the seeds had largely transformed into silver nanoplates after 48 h of LED irradiation.

Moreover, the IPD peak showed a noticeable blueshift. Previous studies have shown that the position of the IPD peak is closely correlated with the edge-to-thickness ratio of the nanoprisms [4,15]. Therefore, the clear observation of the OPQ peak at 48 h suggests a significant contribution of nanoplate thickness to the overall surface plasmon resonance behavior, which could account for the blueshift of the IPD peak.

After 72 and 96 h, the OPQ and IPQ peaks remained stable at 333 and 440 nm, respectively, indicating that the transformation of silver seeds into nanoplates had reached equilibrium. The IPD peak continued to blueshift slightly (to 604 nm and 600 nm, respectively), but no significant changes were observed in the overall UV–vis spectral profiles. Based on these results, we conclude that 72 h represents the optimal irradiation time for the complete transformation of silver seeds into silver nanoplates in this study.

**Figure 3:**
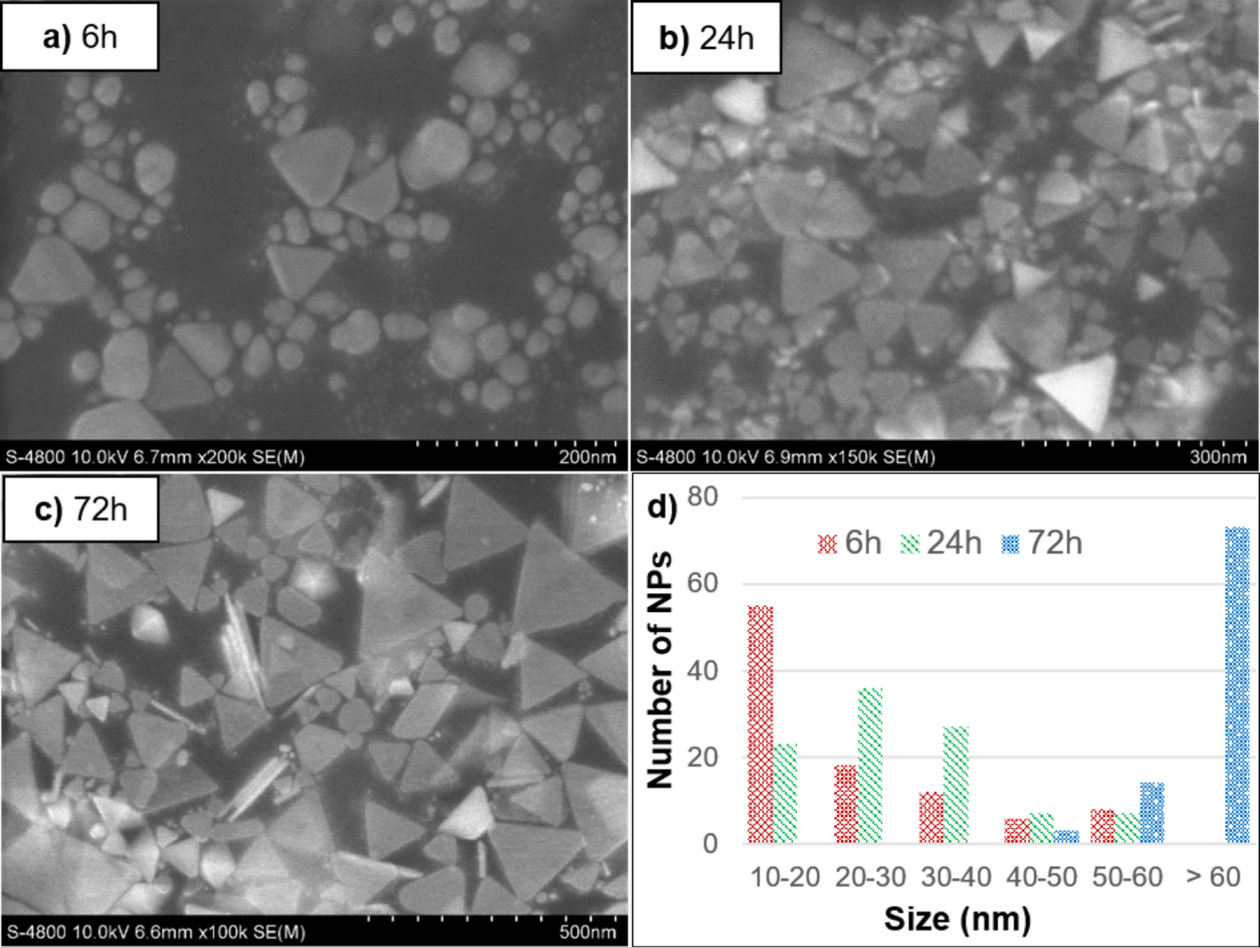
FESEM images of silver nanoparticles after (a) 6 h, (b) 24 h, and (c) 72 h of LED irradiation, and (d) histogram of their size distributions.

From the FESEM images and particle size distribution graph after 12 h of LED irradiation in Figure 3, it is observed that the particle sizes are primarily in the 10–20 nm range, which corresponds to spherical silver nanoparticles. Additionally, some particles with larger sizes of 35–40 nm were also present; these could be AgNPrs or round silver nanoplates, although their number was minimal. This indicates that after 12 h, the sample mainly consists of spherical silver nanoparticles, while nanoplates have started to form but remain scarce. These FESEM results are consistent with the UV–vis spectra discussed previously.

Upon increasing the irradiation time to 48 h, the particle sizes became larger compared to the 12 h sample, predominantly in the range of 25–35 nm, suggesting a greater presence of AgNPrs. However, the persistence of AgNPs and round silver nanoparticles implies that the conversion efficiency from AgNPs to AgNPrs remains relatively low.

After 72 h of LED irradiation, the particle size increased significantly compared to the 12 h and 48 h samples, mostly ranging between 70–80 nm. At this stage, the majority of particles are identified as AgNPrs. Only a negligible number of sAgNPs and round silver nanoplates remain. The average size of the AgNPrs at this stage is 78 nm, approximately double that of the 12 h and 48 h samples. It can thus be concluded that 72 h is the optimal duration for the transformation of AgNPs into AgNPrs using green LED irradiation, aligning well with the UV–vis spectroscopy results.

**Figure 4:**
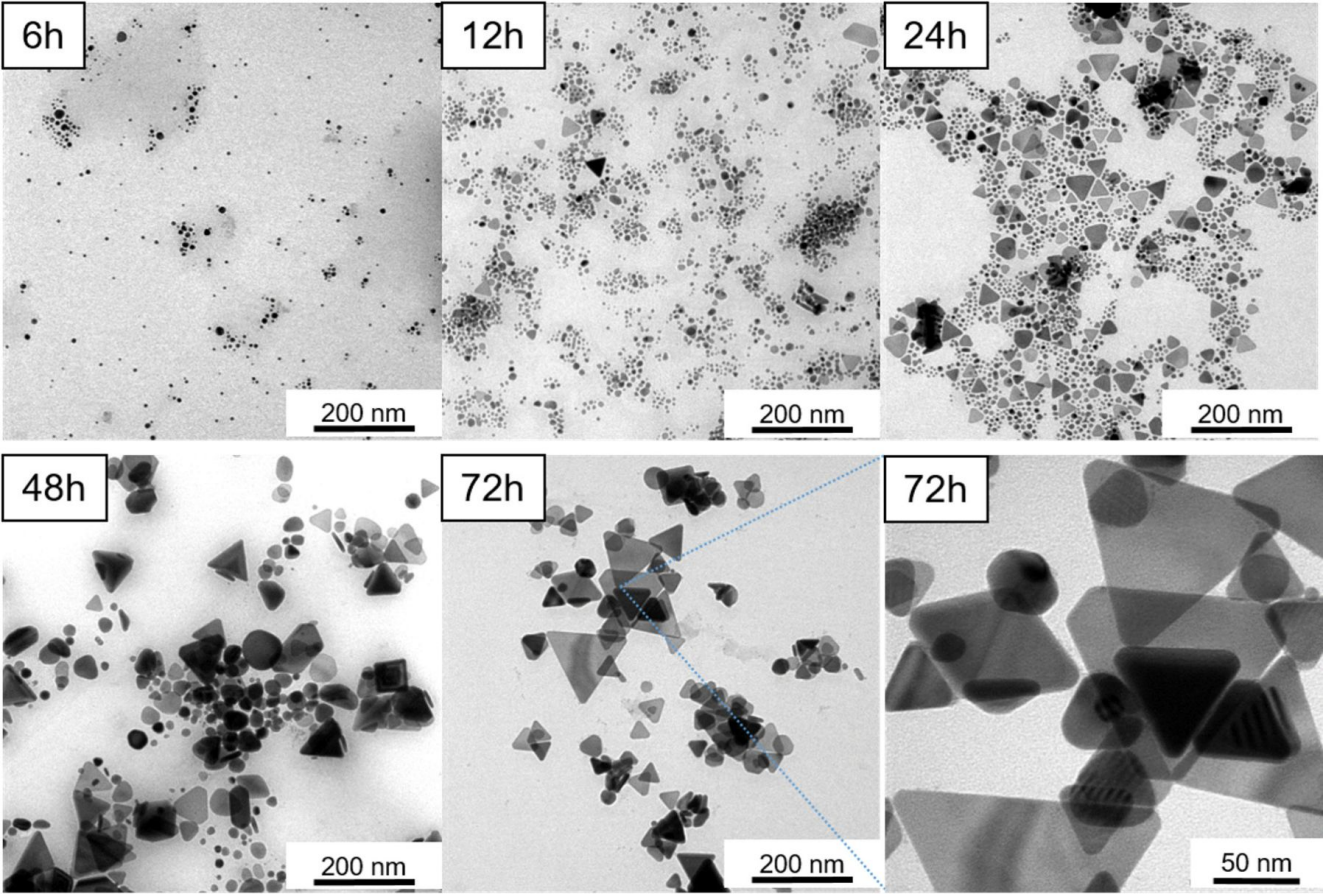
TEM images of silver nanoparticles after 12, 24, 48, and 72 h of LED irradiation.

TEM images of the samples at a magnification of 50,000 after 6, 12, 24, 48, and 72 h of LED irradiation are shown in Figure 4. The results indicate that after 6 h, the sample predominantly contained spherical silver nanoparticles. As the LED irradiation time increased, the proportion of AgNPrs became more significant. After 24 h, both AgNPs and AgNPrs coexisted, which is consistent with the UV–vis spectrum showing two distinct IPD peaks corresponding to each morphology.

After 48 h, AgNP seeds were no longer visibly present in significant amounts; the sample was mainly composed of AgNPrs. This observation correlates with the appearance of the OPQ peak, characteristic of nanoplate thickness, and the disappearance of the IPD peak of AgNPs in the UV–vis spectrum in Figure 2. After 72 h, TEM images showed exclusively AgNPrs, which were clearly visible and well defined, in agreement with the corresponding FESEM images.

**Figure 5:**
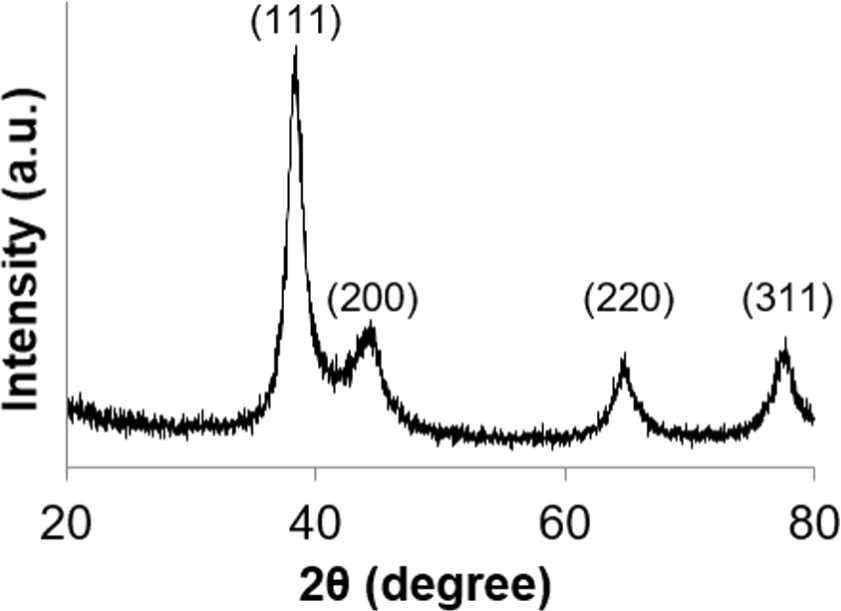
XRD spectrum of AgNPrs after 72 hours of LED irradiation.

The XRD spectrum of AgNPrs after 72 h of LED irradiation (Figure 5) exhibited four distinct broad diffraction peaks at 38.2°, 44.3°, 64.5°, and 77.7°, corresponding to the (111), (200), (220), and (311) crystal planes, respectively. These peaks are characteristic of the face-centered cubic (fcc) structure of silver nanoparticles. The diffraction intensity at the (111) crystal facets was three times higher than that at the (200) facets, consistent with the typical structure of AgNPrs [9,16]. Moreover, no diffraction peaks corresponding to Ag_2_O were detected, indicating that the photoreduction process for synthesizing AgNPrs was efficient.

In summary, after 72 h of LED irradiation under the experimental conditions, small spherical silver nanoparticle seeds had been almost completely converted into silver nanoprisms. Unlike conventional chemical synthesis methods, the photochemical LED-based approach does not produce fragmented or etched structures, resulting in well-formed AgNPrs. Additionally, this method avoids excessive oxidative–reductive agents such as H_2_O_2_, thereby improving the structural integrity, stability, and potential applicability of the resulting AgNPrs.

### Formation mechanism of AgNPrs

During the seed development process leading to the formation of AgNPrs, two critical components play decisive roles, namely, the source of silver ions (Ag^+^) and the reducing agent that converts Ag^+^ to metallic silver (Ag^0^).

Upon LED irradiation of the system containing initial AgNPs seeds, the atomic layers of silver at the nanoparticle surfaces are excited and oxidized by dissolved oxygen in the solution, generating Ag^+^ ions [15]. These ions serve as the source of Ag^+^ for the seed growth process. TSC preferentially binds to the (111) crystal facets of silver nanoparticles. Under LED illumination, TSC is excited and oxidized, acting as a reducing agent through the following reactions:


[1] $${\text{citrate}}^{3-}+h\nu \to \text{acetone-}1,3{\text{-dicarboxylate}}^{2-}+{\text{CO}}_{2}+{\text{e}}^{-}$$

[2] $${\text{Ag}}^{+}+{\text{e}}^{-}\to {\text{Ag}}^{0}$$

**Figure 6:**
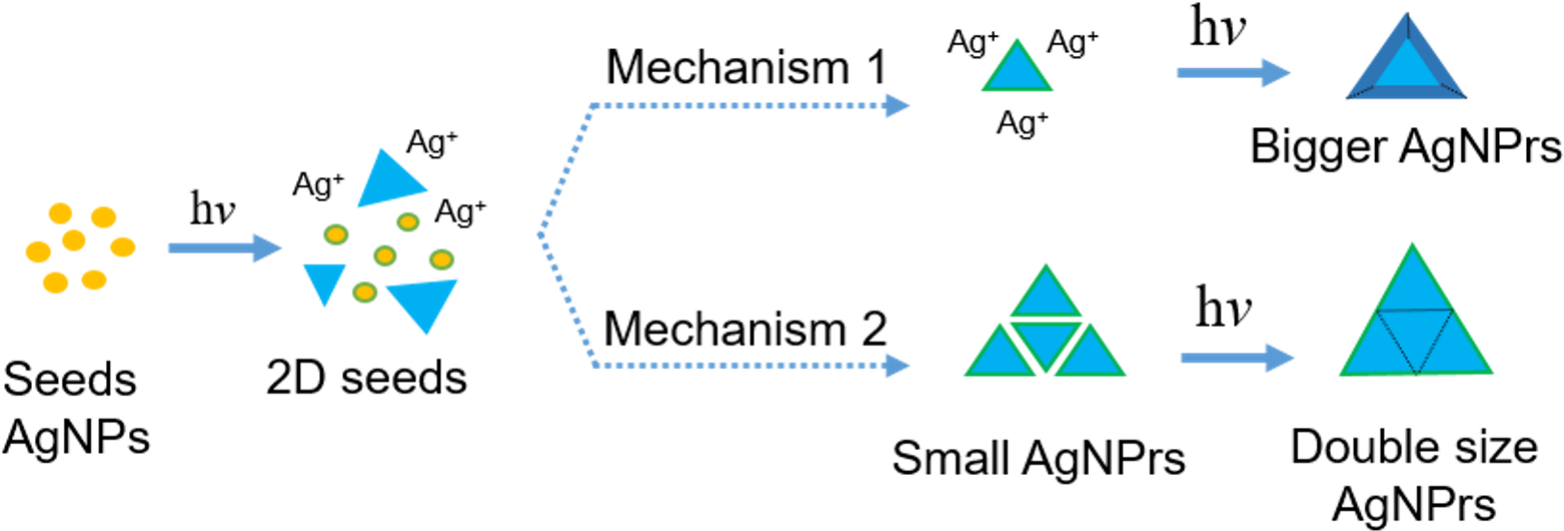
Mechanisms proposed for AgNPrs formation.

Electrons released from TSC immediately reduce Ag^+^ to Ag^0^. Since the (111) facets are protected by TSC, the newly formed Ag^0^ preferentially deposits on the (100) and (110) surfaces, leading to planar two-dimensional growth and the formation of planar twinned seeds [6–7]. Subsequently, as LED irradiation continues, the surfaces of the silver nanomaterials (initial AgNP seeds, planar twinned seeds) are continuously excited, and the growth of AgNPrs proceeds via two mechanisms (Figure 6). These mechanisms are: (1) TSC continues to be excited and promotes the reduction of Ag^+^, resulting in the deposition of Ag^0^ on the edges of small AgNPrs, thereby increasing their edge lengths over time [7,10,16]. (2) Instead of further edge growth, small AgNPrs can laterally attach to one another. At the junctions, Ag^+^ is reduced by TSC under LED stimulation, facilitating their fusion and leading to the formation of larger AgNPrs with approximately double the original edge length [16–17].

In the current study, the high precursor concentration (1 mM AgNO_3_) likely promoted frequent interparticle collisions. Initially, AgNPrs formed via mechanism 1 and grew to approximately 40 nm in size. Subsequently, these smaller AgNPrs underwent edge-to-edge fusion, yielding larger AgNPrs with edge lengths exceeding 80 nm, consistent with mechanism 2.

### SERS properties of silver nanoparticles

In this study, 4-MBA was used as the probe molecule due to its wide use in both intrinsic and extrinsic SERS applications [18–19]. It exhibits strong affinity toward silver nanoparticles via Ag–S bonding, which significantly enhances the SERS signal. Additionally, the carboxylic acid (–COOH) group of 4-MBA readily enables further functionalization with other compounds, making it suitable for biosensing applications.

**Figure 7:**
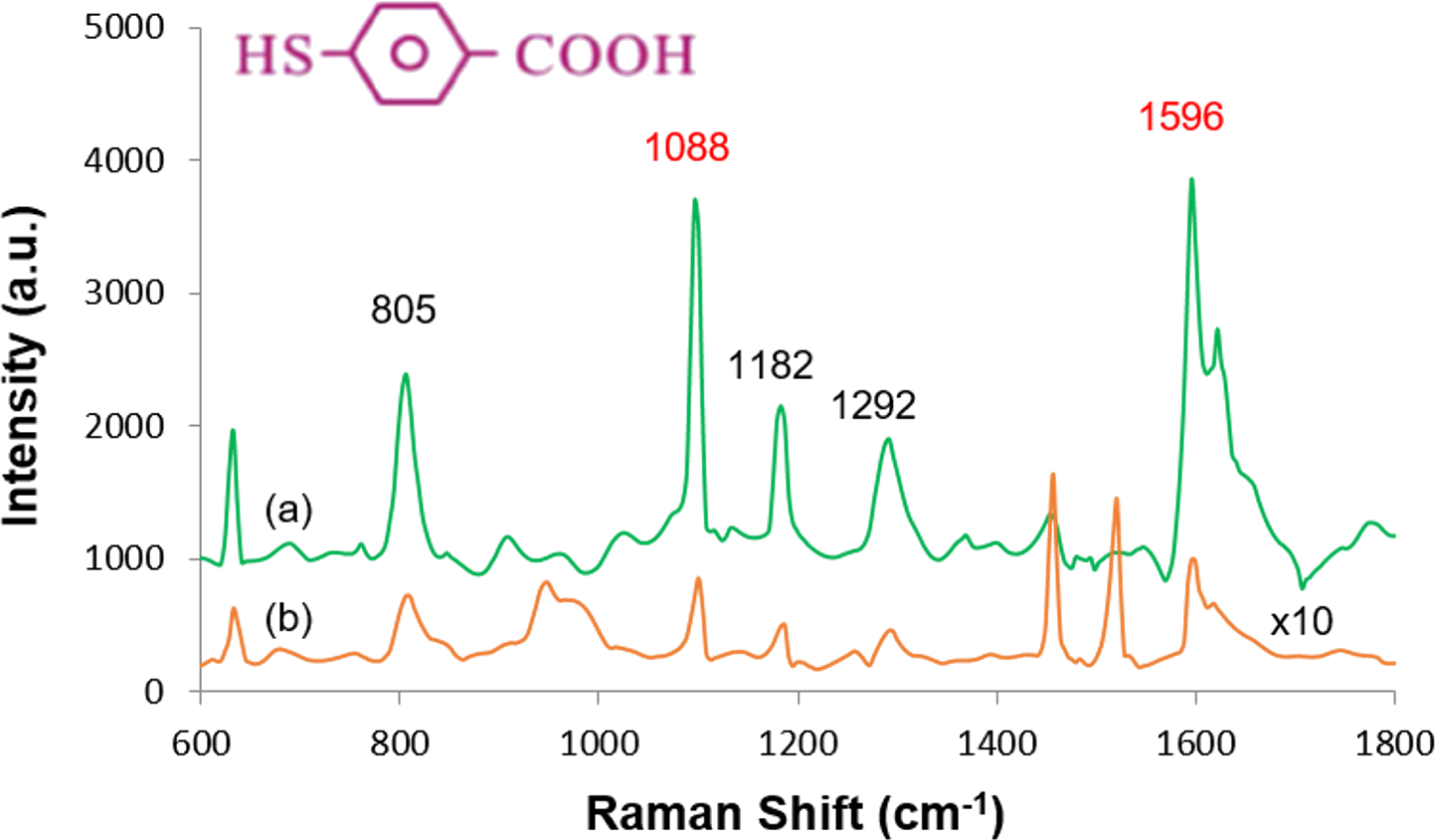
Raman spectra of (a) solid 4-MBA and (b) 4-MBA (10^−1^ M, amplified tenfold).

The Raman spectrum of solid 4-MBA (Figure 7a) displays two dominant peaks at 1088 and 1596 cm^−1^, corresponding to the stretching vibrations of the aromatic ring. Weaker peaks are observed at 1182 and 1292 cm^−1^, attributed to C–H stretching, and at 805 cm^−1^, corresponding to the bending vibration of the carboxylate group (COO^−^) [20–21]. The Raman spectrum of a 100 mM 4-MBA solution, recorded on a Si wafer and amplified tenfold (Figure 7b), also shows the 1088 and 1596 cm^−1^ peaks with lower intensity, consistent with literature reports at the same concentration and excitation wavelength of 532 nm [20].

**Figure 8:**
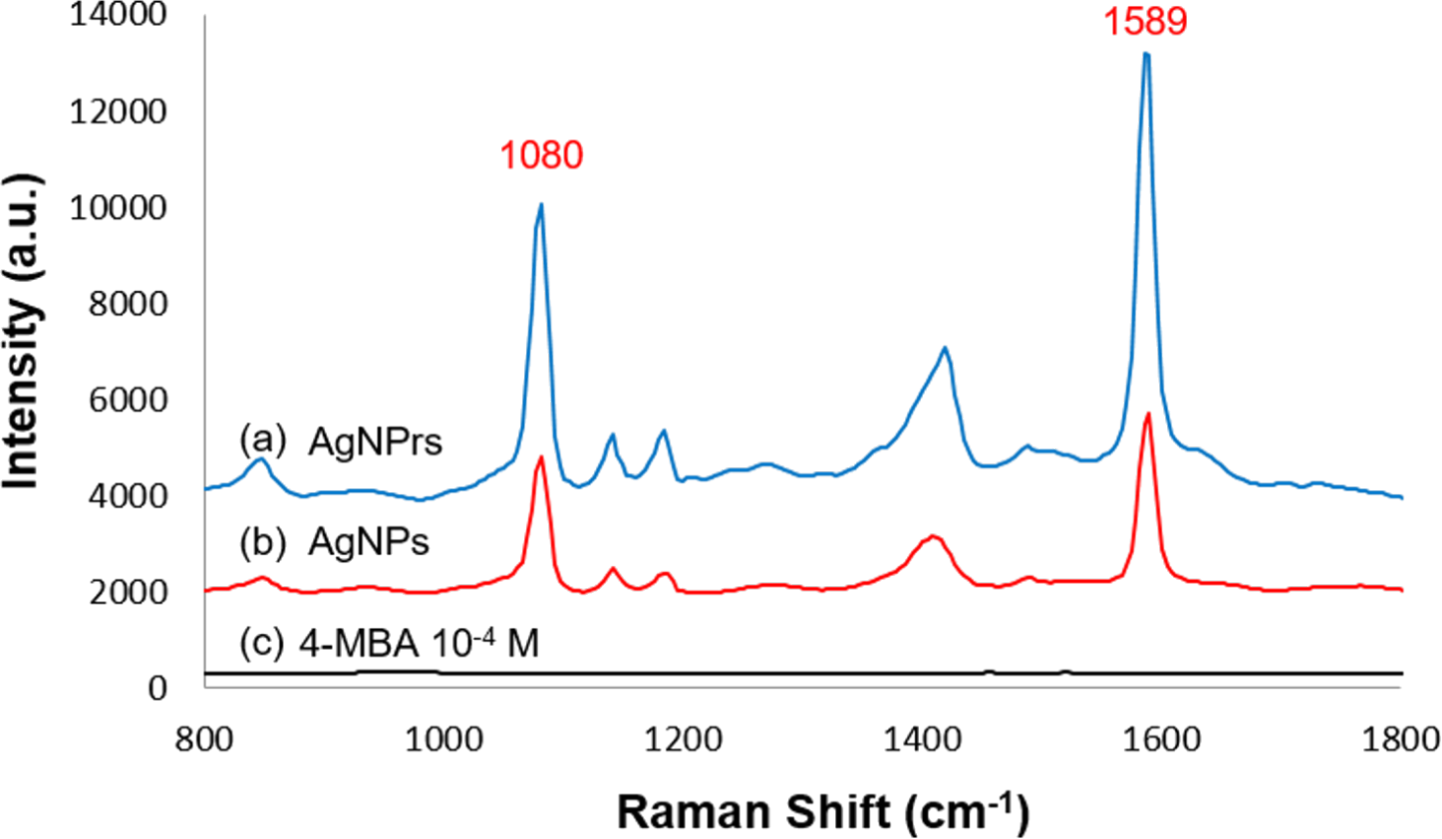
Raman spectra of 4-MBA (10^−5^ M) with either (a) AgNPrs (after 72 h of LED irradiation) or (b) AgNPs (seeds), as well as of (c) 4-MBA 10^−4^ M.

The Raman spectrum of 4-MBA at 10^−4^ M (Figure 8c) shows no detectable peaks, whereas the SERS spectrum of 4-MBA at 10^−5^ M, enhanced by both AgNPs (Figure 8b) and AgNPrs (Figure 8a), exhibit strong signals at 1080 and 1589 cm^−1^, corresponding to aromatic ring vibrations. Additional weaker peaks at 1148 and 1184 cm^−1^ are assigned to C–H stretching modes [21–22]. These findings demonstrate the SERS-enhancing capability of the silver nanomaterials synthesized in this study.

The SERS enhancement effect of each nanomaterial was evaluated based on the intensity of the 1589 cm^−1^ peak. Overall, AgNPrs exhibited significantly stronger SERS activity compared to AgNPs (seeds). The measurements were performed under identical experimental conditions, including fixed laser polarization, to ensure consistency and the current comparison is intended to be qualitative in nature.

The enhancement factor (EF) was calculated using Equation 3 [23]:


[3] $$\text{EF}=\frac{I_{\text{SERS}}\cdot N_{\text{bulk}}}{I_{\text{bulk}}\cdot N_{\text{SERS}}},$$

where *I*_SERS_ and *I*_bulk_ are the Raman intensities of 4-MBA in the presence and absence of silver nanomaterials, respectively. *N*_SERS_ and *N*_bulk_ are the number of 4-MBA molecules excited by the laser under SERS and non-SERS conditions, respectively. Since the same volume (20 µL) and method were used, *N*_SERS_ and *N*_bulk_ can be substituted by *C*_SERS_ and *C*_blank_, corresponding to the concentrations of 4-MBA [22]. In this study, *C*_blank_ = 10^−1^ M and *C*_SERS_ = 10^−5^ M. Based on this equation, the enhancement factors for AgNPrs and AgNPs were calculated to be 1.15 × 10^6^ and 4.2 × 10^5^, respectively. These results confirm that AgNPrs exhibited superior SERS performance, consistent with prior studies investigating the direct SERS detection of 4-MBA [18–19].

Due to their anisotropic structure, AgNPrs concentrate surface electrons and form electromagnetic “hot spots” at the edges and tip of the nanoprisms [22,24–25]. These hot spots significantly enhance the local electric field, thereby improving SERS efficiency relative to isotropic spherical nanoparticles. For AgNPrs, hot spots at the tip and edges play a key role in SERS enhancement, while the flat surfaces contribute less [25]. Consequently, AgNPrs with triangular sharp, well-defined vertices exhibit better SERS properties than round-edged or poorly defined nanoplate structures.

**Figure 9:**
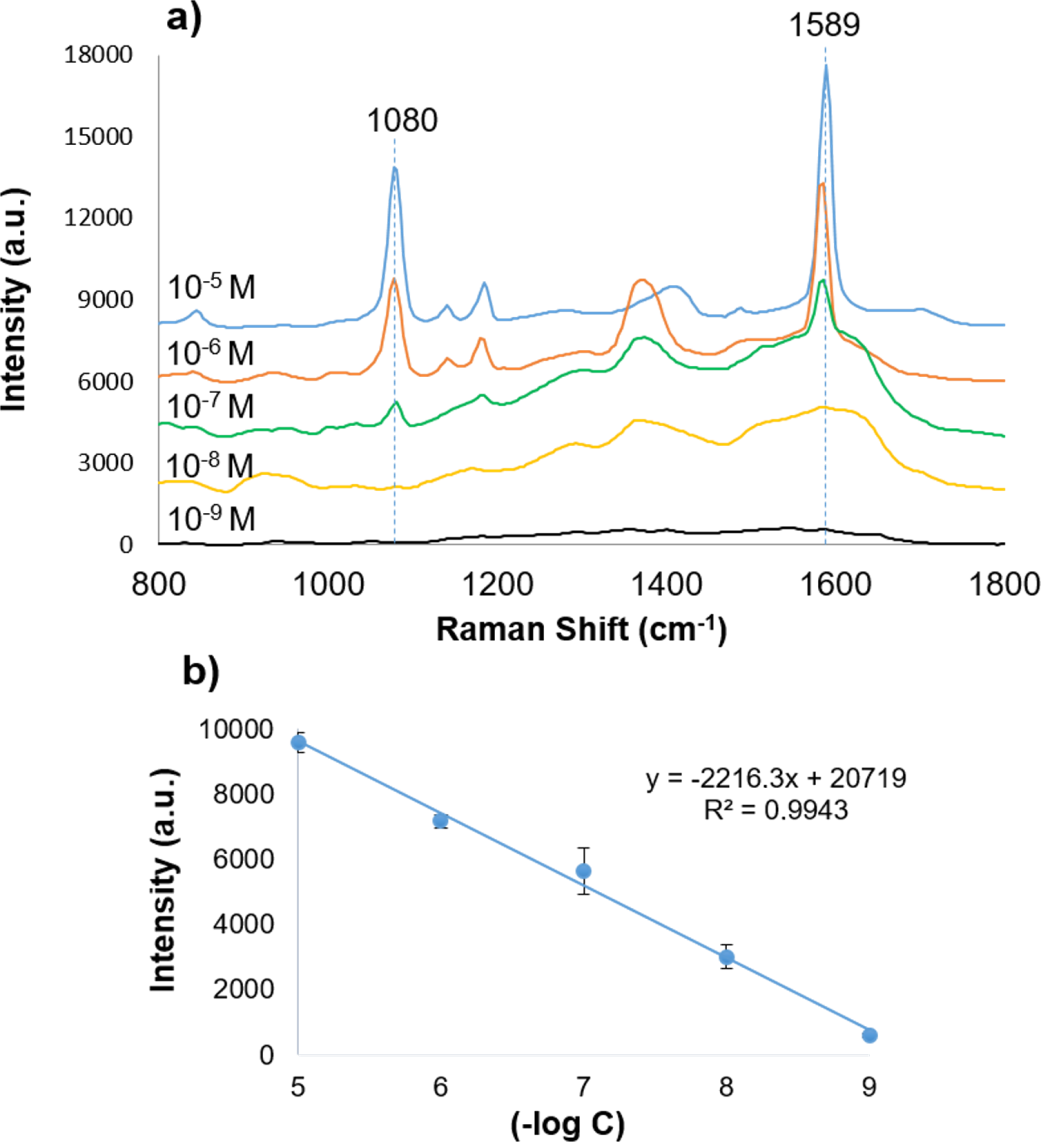
(a) Raman spectra of the mixture of AgNPrs and 4-MBA with various concentrations and (b) relationship between the intensity of peak at 1589 cm^−1^ and the concentration of 4-MBA in the range from 10^−9^ to 10^−5^ M, each data point represents the average value from six independent measurements.

To further evaluate sensitivity, Raman spectra of mixture of AgNPrs and 4-MBA with various concentration ranging from 10^−5^ M to 10^−9^ M were recorded. As shown in Figure 9a, the peak at 1589 cm^−1^ gradually decreased in intensity with decreasing concentration. Even at 10^−7^ M, the peak remained clearly observable. At 10^−8^ M and 10^−9^ M, the peak intensity was still detectable but less well-defined. This is likely due to the fixed quantity of AgNPrs across all experiments, while the concentration of 4-MBA decreased logarithmically. Since the sample was prepared by drop casting onto a Si wafer, AgNPrs tended to aggregate, which may have led to reduced resolution of the Raman peaks at very low analyte concentrations.

The relationship between 4-MBA concentration and the intensity of the 1589 cm^−1^ peak (Figure 9b) was found to be linear, following the equation *y* = −2216.3*x* + 20719, *R*^2^ = 0.9943. This linear correlation indicates that the lowest 4-MBA concentration detectable with this AgNPr-based SERS substrate was 10^−9^ M, confirming its potential for ultrasensitive SERS-based sensing applications.

## Conclusion

This study demonstrates a facile, eco-friendly photochemical method for synthesizing AgNPrs using green LED irradiation without the need for harsh chemical reagents. The transformation of AgNPs seeds into AgNPrs was confirmed through UV–vis, FESEM, and TEM analyses, with optimal formation achieved after 72 h of irradiation. The resulting AgNPrs showed significantly enhanced SERS performance compared to spherical AgNPs, attributed to the anisotropic morphology and formation of electromagnetic hot spots at the nanoplate tips and edges. Using 4-MBA as a Raman probe, the AgNPr-based SERS substrate enabled detection at concentrations as low as 10^−9^ M with a strong linear response. These results highlight the potential of LED-synthesized AgNPrs as high-performance, low-cost, and environmentally benign substrates for sensitive SERS applications in chemical and biosensing fields.

## Experimental

### Chemical

Silver nitrate (AgNO_3_, >99%), trisodium citrate tribasic dihydrate (TSC, >99%), 4-mercapto benzoic acid (4-MBA, 99%) were obtained from Sigma-Aldrich, Darmstadt, Germany. ʟ-arginine (L-A, 99%), sodium borohydride (NaBH_4_, 98%) were obtained from Merck, Darmstadt, Germany. Polyvinylpyrrolidone (PVP K30) were purchased from Prolabo, Kennersburg, NJ, USA. Chemicals were used without any purification. Deionized (DI) water with a resistance of 18 MΩ·cm (Milli-Q) was used in all the experiments. All glasswares were cleaned with aqua regia before use.

#### Synthesis of seeds

A total of 10 mL of 0.05 M trisodium citrate (TSC), 300 µL of 0.05 M polyvinylpyrrolidone (PVP), 20 mL of 5 mM AgNO_3_, and 500 µL of 0.005 M ʟ-arginine solution were added into a beaker. Deionized water was then added to adjust the total volume of the solution to 100 mL. The mixture was magnetically stirred at 500 rpm for 5 min. Subsequently, 1.6 mL of 100 mM NaBH_4_ solution (cooled with ice) was rapidly injected using a micropipette. The solution was stirred for additional 30 min, during which the color of the mixture changed from colorless to dark yellow, then to bright yellow, and finally to a lighter yellow. The solution was stirred at room temperature and aged in the dark overnight.

#### Manufacturing of AgNPrs

A volume of 20 mL of the seed solution was transferred into 25 mL glass vials (Wheaton, Germany). These vials were placed vertically at a fixed distance of 8 cm from the LED light source, inside a cardboard box internally lined with aluminum foil. The LEDs were oriented perpendicularly to the vials to ensure that each vial was consistently positioned at the center of the incident light beam throughout all experiments. The seed solutions were irradiated with green LEDs (520 ± 20 nm, Epistar 10 W chip, Taiwan) for 6, 12, 24, 48, and 72 h. During the irradiation process, the color of the solution gradually shifted from pale yellow to green and eventually to blue.

#### Characterization

The optical properties of the samples were characterized using UV–vis spectroscopy (Jasco V-670) with a scanning range from 900 to 200 nm. Samples were diluted tenfold with deionized water prior to measurement.

To determine the morphology, particle size of the synthesized nanostructures, the samples were analyzed using field-emission scanning electron microscopy (FESEM, Hitachi S-4800, Japan) and transmission electron microscopy (TEM, JEM-1400, Japan). For TEM analysis, a droplet of the nanoparticle dispersion was deposited onto a 3 mm copper grid and allowed to dry at room temperature. For FESEM analysis, the dried sample was mounted on conductive carbon tape and imaged at an accelerating voltage of 10 kV. TEM imaging was performed at an accelerating voltage of 100 kV.

The structure of the AgNPrs was analyzed by X-ray diffraction (XRD) using a Bruker D8 Advance instrument equipped with a Cu Kα radiation source (40 kV, 40 mA) at a scanning rate of 4°/min.

#### SERS measurements

The SERS properties of the AgNPs and AgNPrs were investigated on a Si wafer surface with the 4-MBA Raman reporter. The Si wafer was cut to a size of 1 × 1 cm and treated with piranha solution (H_2_SO_4_/H_2_O_2_ = 3:1) before used to remove organic compounds. A 2 mL sample was centrifuged at 12,000 rpm for 15 min, with the supernatant discarded, and the solid residue was re-dispersed in 1 mL of distilled water. 50 µL of 10^−4^ M 4-MBA solution was added to 450 µL of the re-dispersed sample and left for 1 h at room temperature. 20 µL of the resulting mixture was placed on the Si wafer substrate, allowed to dry at room temperature, and Raman measurements were performed (HORIBA XploRA ONE TM, France) using a 532 nm laser source and a 10× magnification microscope. Measurements were taken at ten random positions, and the average intensity was recorded.

## Supporting Information

File 1 Additional figures and tables.

## Data Availability

Data generated and analyzed during this study is available from the corresponding author upon reasonable request.
