# Supplementary material for: Photochemical synthesis of silver nanoprisms via green LED irradiation and evaluation of SERS activity
Source: Beilstein J Nanotechnol. 2025 Aug 26;16:1417–27. doi: 10.3762/bjnano.16.103 (PMC12415907; doi:10.3762/bjnano.16.103)
Supplement: File 1 — Additional figures and tables. [file Beilstein_J_Nanotechnol-16-1417-s001.pdf]

BEILSTEIN JOURNAL OF NANOTECHNOLOGY

Supporting Information

for

Photochemical synthesis of silver nanoprisms via green LED irradiation and evaluation of SERS activity

Tuan Anh Mai-Ngoc, Nhi Kieu Vo, Cong Danh Nguyen, Thi Kim Xuan Nguyen and Thanh Sinh Do

Beilstein J. Nanotechnol. **2025,** *16,* 1417–1427. doi:10.3762/bjnano.16.103

Additional figures and tables

License and Terms: This is a supporting information file under the terms of the Creative Commons Attribution License (https://creativecommons.org/licenses/by/4.0). Please note that the reuse, redistribution and reproduction in particular requires that the author(s) and source are credited and that individual graphics may be subject to special legal provisions.
The license is subject to the *Beilstein Journal of Nanotechnology* terms and conditions: (https://www.beilstein-journals.org/bjnano/terms)

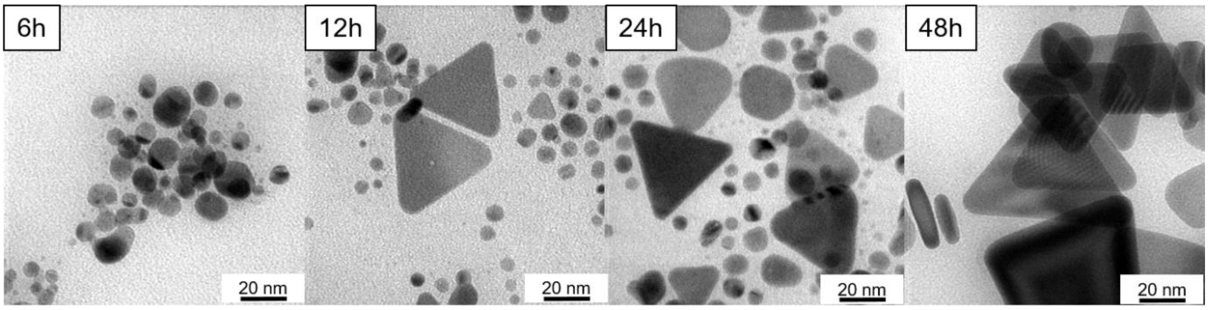

Figure S1: TEM images of the samples at a magnification of 200,000 after 6, 12, 24 and 48 hours of LED irradiation.

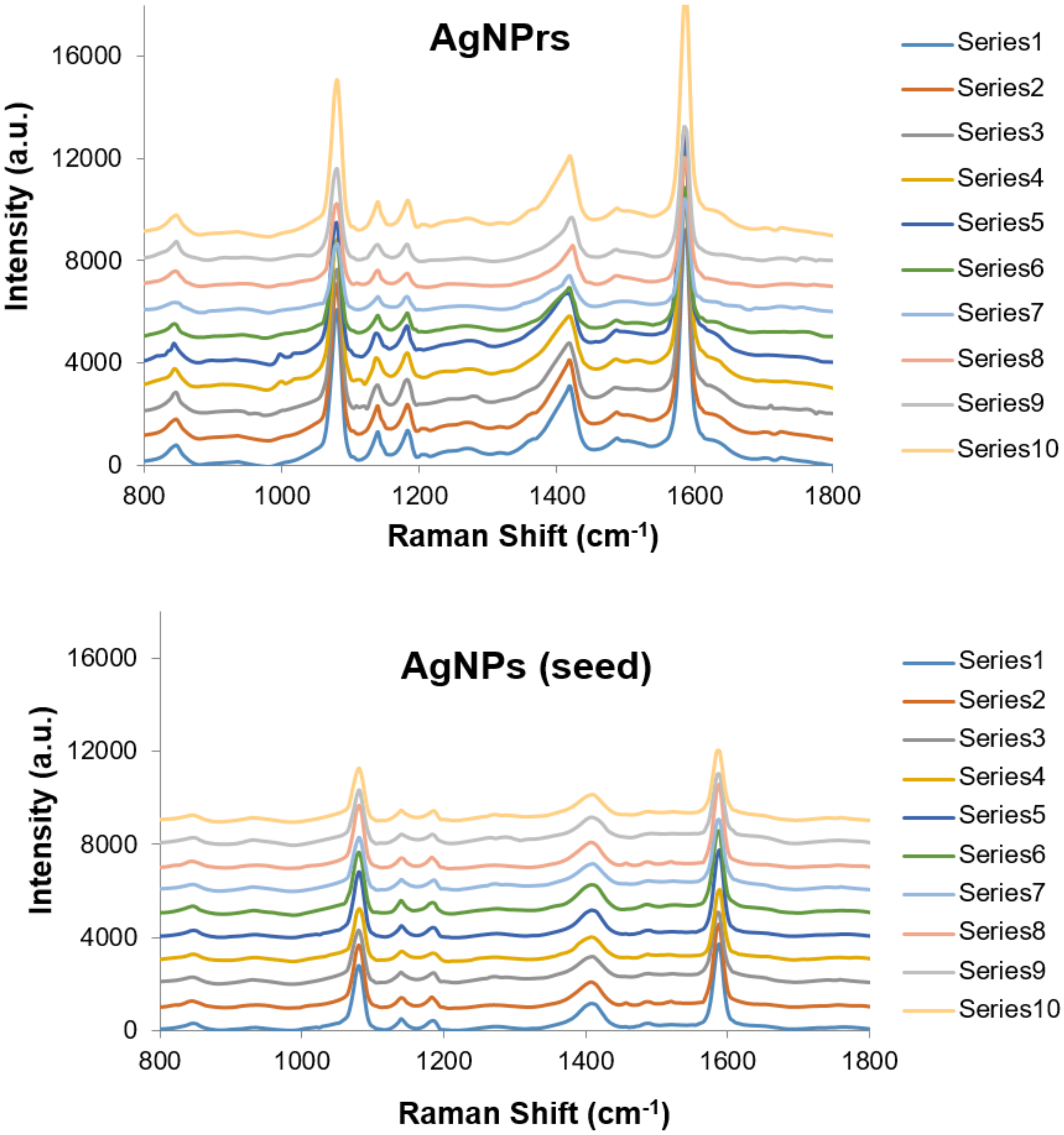

Figure S2: SERS spectra of 4-MBA at 10^{-5} M, enhanced by (a) AgNPrs and (b) AgNPs, recorded 10 different measurements.

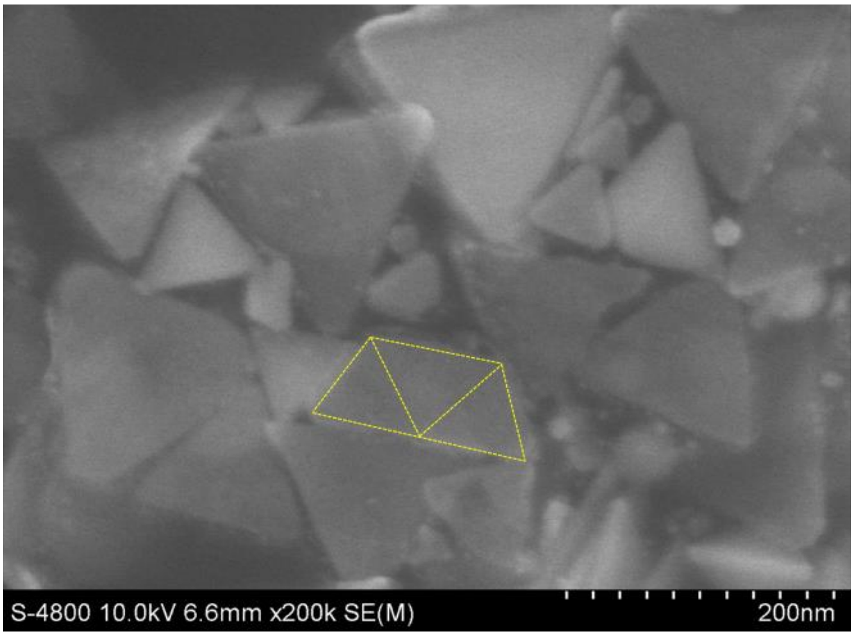

Figure S3: FE-SEM image of AgNPrs at 72 h LED irradiation: a trapezoid-shaped silver nanoparticles was seen. An additional small AgNPrs is required to assemble an AgNPrs with twice the original size (via Mechamism 2 in Figure 6).

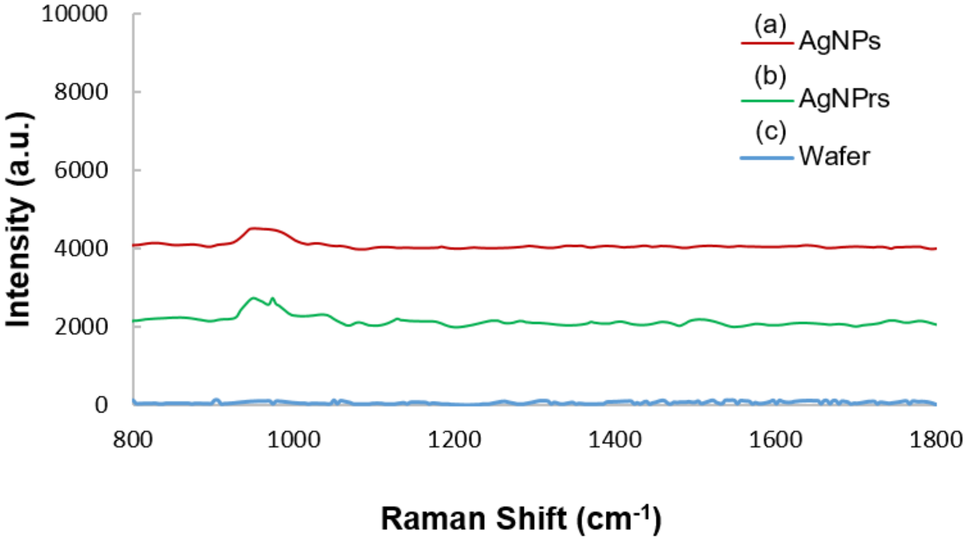

Figure S4: Raman spectra of (a) AgNPrs, (b) AgNPs an (c) wafer.

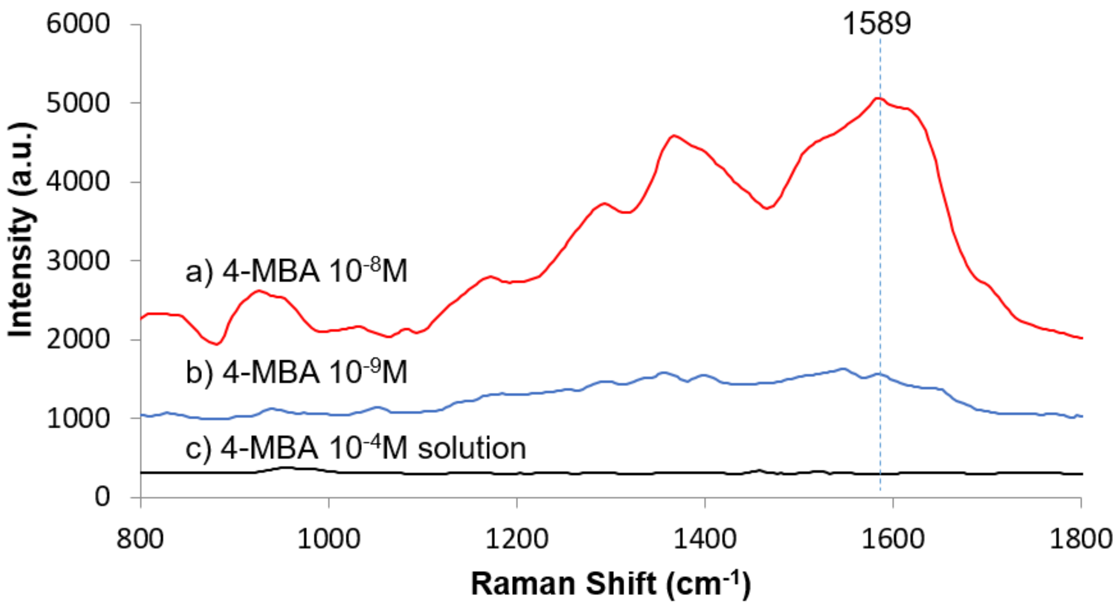

Figure S5: SERS spectra of (a) 4-MBA 10^{-8} M, (b) 4-MBA 10^{-9} M and (c) Raman spectrum of 4-MBA 10^{-4} M (not enhanced by AgNPrs).

Table S1: UV–vis absorption peak of silver nanoparticles at different LED irradiation times.

Samples	Peak OPQ (nm)	Peak IPQ (nm)	Peak IPD (nm)
**6 h**	-	-	402
**12 h**	-	-	405<br>650
**24 h**	-	-	405<br>662
**48 h**	333	420	654
**72 h**	333	440	604
**96 h**	333	440	600
